# Pharmacological and genomic profiling of neurofibromatosis type 1 plexiform neurofibroma-derived schwann cells

**DOI:** 10.1038/sdata.2018.106

**Published:** 2018-06-12

**Authors:** Marc Ferrer, Sara J. C. Gosline, Marigo Stathis, Xiaohu Zhang, Xindi Guo, Rajarshi Guha, Dannielle A. Ryman, Margaret R. Wallace, Laura Kasch-Semenza, Haiping Hao, Roxann Ingersoll, David Mohr, Craig Thomas, Sharad Verma, Justin Guinney, Jaishri O. Blakeley

**Affiliations:** 1 National Center for Advancing Translational Sciences (NCATS), Division of Pre-clinical Innovation, National Institutes of Health, Bethesda, MD, USA; 2 Sage Bionetworks, Seattle, WA, USA; 3 Neurofibromatosis Therapeutic Acceleration Program (NTAP), Department of Neurology, Johns Hopkins University School of Medicine, Baltimore, MD, USA; 4 Department of Molecular Genetics & Microbiology, University of Florida College of Medicine, Gainesville, FL, USA; 5 Genetic Resources Core Facility (GRCF), Johns Hopkins School of Medicine, Baltimore, MD, USA; 6 Deep Sequencing & Microarray Core, Molecular Biology and Genetics, Johns Hopkins School of Medicine, Baltimore, MD, USA; 7 Center for Inherited Disease Research (CIDR), Johns Hopkins University School of Medicine, Baltimore, MD, USA

**Keywords:** Assay systems, High-throughput screening, Cancer genomics

## Abstract

Neurofibromatosis type I (NF1) is an autosomal dominant genetic condition characterized by peripheral nervous system tumors (PNSTs), including plexiform neurofibromas (pNFs) that cause nerve dysfunction, deformity, pain damage to adjacent structures, and can undergo malignant transformation. There are no effective therapies to prevent or treat pNFs. Drug discovery efforts are slowed by the ‘benign’ nature of the Schwann cells that are the progenitor cells of pNF. In this work we characterize a set of pNF-derived cell lines at the genomic level (via SNP Arrays, RNAseq, and Whole Exome- Sequencing), and carry out dose response-based quantitative high-throughput screening (qHTS) with a collection of 1,912 oncology-focused compounds in a 1536-well microplate cell proliferation assays. Through the characterization and screening of *NF1*^−/−^, *NF1*^*+/+*^ and *NF1*^+/−^ Schwann cell lines, this resource introduces novel therapeutic avenues for the development for *NF1* associated pNF as well as all solid tumors with *NF1* somatic mutations. The integrated data sets are openly available for further analysis at http://www.synapse.org/pnfCellCulture.

## Background & Summary

Neurofibromatosis type I (NF1) is an autosomal dominant genetic syndrome characterized by multiple peripheral nervous system tumors (PNST), including plexiform neurofibromas (pNF)^1^. The prevalence of NF1 is estimated at 1/3000 and roughly 30–50% of people with NF1 develop pNF^2,3^. pNFs are complex nerve sheath tumors that often cause physical deformity, neurologic disability and can transform into aggressive sarcomas called malignant peripheral nerve sheath tumors (MPNST)^2–4^.

Therapeutic development for pNFs has been challenging because pNFs are histologically benign and slow, but relentless in their growth. In addition, they are composed of multiple cell types including Schwann cells, fibroblasts, mast cells, macrophages, neuronal axons, perineurial cells and extracellular matrix materials such as collagen^5–7^. However, multiple lines of evidence point to the *NF1* ^−/−^ Schwann cell as the tumor initiating cell and therefore the priority target cell for drug development^5–9^.

The syndrome of NF1 is associated with constitutional heterozygosity for *NF1.* Plexiform neurofibromas develop when there is a second, somatic mutation resulting in *NF1*^−/−^ Schwann cells^10,11^. Given this, ideal therapies will target *NF1*^−/−^ Schwann cells, but not *NF1*^*+/−*^ cells to yield maximum anti-tumor specificity. The lack of proven effective therapies to date is partially due to their clinical and cellular heterogeneity as well as the relative scarcity of preclinical model systems to test potential therapies^12,13^. In part, this is because historically culturing of both normal and *NF1* mutant Schwann cells was difficult. Recently, advances in isolating Schwann cells from human tissues, immortalization techniques, and culturing conditions have resulted in the creation of viable *NF1* cell lines for pharmacological interrogation^11,14–16^.

To accelerate the discovery of key signalling pathways for pharmacologic targeting, a large set of immortalized, NF1 patient derived Schwann cells representing the genetic diversity of human plexiform tumors was screened in dose response quantitative high throughput screens (qHTS) format. Here, we report on the development of a 1536-well qHTS compatible cell proliferation assays with *NF1*^−/−^, *NF1*^+/−^ and *NF1*^+/+^ Schwann cells, and their use for implementation in screening a collection of oncology drugs of the NCATS Mechanism Interrogation PlatE (MIPE 4.0) library^17^. The MIPE 4.0 library is comprised of 1912 small molecules with diverse mechanism of action; 40% are approved drugs, 20% consist of phase I-III investigational drugs, and 40% consist of preclinical molecules. For a full list of molecules see Table syn11699007 (Data Citation 1), where the compounds are annotated with their nominal targets, when known. Redundancy of compounds targeting the same putative target allow target-based enrichment response analysis and identification of pathway-based patterns of pharmacological responses across cell lines. Screening this library with human patient-derived pNF Schwann cells could yield important insights, including: (1) identification of approved drugs which could be repurposed for pNF therapy, (2) discovery of new targets not previously considered for the treatment of these tumors and, (3) investigation of drug combinations with a focus on differential response between *NF1*^−/−^ cells and either haploinsufficient or normal (wild-type) cells.

Through detailed characterization of both primary and immortalized *NF1*^−/−^, *NF1*^*+/−*^, *NF1*^*+/+*^ Schwann cells with SNP Array, RNAseq, and Whole Exome Sequencing, we confirm that the immortalized cell lines reliably represent primary human cells in the drug screening platform, validating a new scientific resource. These data, together with the data from the 1536-well cell proliferation assays, represent novel tools to facilitate therapeutic discovery for *NF1* mutant driven rare and treatment resistant tumors.

## Methods

### Cell line overview and data collection

**Figure 1:**
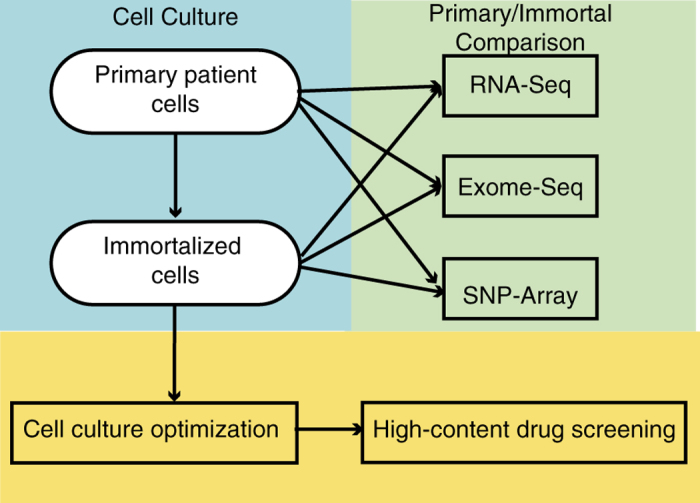
Schema of sample and data collection.

Diverse high throughput data was collected from cell lines cultured from plexiform neurofibromas as described in Table 1. The overall process for data collection is depicted in Fig. 1. Immortalized and primary cell lines derived from pNF patient samples were first compared using high throughput genomic and transcriptomic analysis. Then the culture conditions were further optimized so that the immortalized cells could be further profiled using the high-throughput drug-screening assay. A graphical interface to assist with review of these results is located at http://www.synapse.org/pnfCellCulture (Data Citation 2).

### Cell line immortalization

Eleven Schwann cell lines from seven individuals were obtained from the Wallace laboratory as described previously^14^. The six cell lines with the most reliable growth performance in culture were selected for detailed characterization by short tandem repeat (STR) analysis using a PowerPlex 18D System (Table 1). The following sets of samples were each derived from unique individuals: (1) pNF95.6 and ipNF95.6; (2) pNF05.5, ipNF05.5, and ipNF05.5 (6 mixed clones derived from original immortalized cell line); (3) pNF04.4 and ipNF04.4; (4) pnNF95.12B and sipNF95.12B, and (5) pn02.3-2λ and ipn02.3-2λ. All of the ‘95.11’ cells (pNF95.11b C, pnNF95.11c, ipNF95.11b C, and ipnNF95.11c) were derived from a single individual, although though pnNF95.11c and ipnNF95.11c are from heterozygous Schwann cells from the same patient (non-tumor nerve)^14^. This was confirmed by CLA analysis. Three lines (i.e., ipnNF02.8, ipNF06.2 A, and ipNF95.11b C/T) were not forwarded for genomic characterization either due to unpredictable passage senescence or differential transduction methodologies (i.e., pnNF95.11c and ipnNF95.11c were transduced with retrovirus)^14^ that may have contributed to divergent pharmacological responses. For the remaining viable six human Schwann cell lines characterized, all were expanded beyond passage 15 (median 25, range 18-75) and all cells derived from the same patient showed matched allele with loci patterns.

### Cell line culture conditions

Primary pNF cell cultures were established after tissue dissociation and maintained in Gibco Dulbecco's Modified Eagle Medium (DMEM) with 10% fetal bovine serum supplemented with glutamine and neuregulin, on a laminin matrix, as previously described (Muir *et al.*, 2001). Immortalized pNF cells were grown initially in T25 and then transferred into T225 tissue culture flasks in DMEM, high glucose, GlutaMAX™ with 10% Fetal Bovine Serum (FBS) and 1X Penicillin-Streptomycin. DMEM and Penicillin-Streptomycin were purchased from Life Technologies (Grand Island, NY); FBS was purchased from HyClone Laboratories (SH30071.03) South Logan, Utah). Gibco 1X Phosphate-buffered saline (PBS), pH 7.4 and 0.25% Trypsin+EDTA (both purchased from Life Technologies) were used to dissociate and passage cells. For cell passaging and scale up, cells were grown in flasks to approximately 80% confluency, dissociated, measured with a Nexcelom cell counter, diluted 1:100 -fold in cell culture media, and added to a new flask. For HTS testing purposes, the average number of cells needed for screening the MIPE collection was ~20e^6^. Human Foreskin Fibroblasts (HFF) cells were cultured in Gibco Dulbecco's Modified Eagle Medium (DMEM), with 10% Fetal Bovine Serum (FBS) and 1X Penicillin-Streptomycin. All cells were confirmed mycoplasma-free before testing with the MycoAlert™ Mycoplasma Detection Kit from Lonza, used as described by the vendor.

### SNP Array

DNA was extracted from the described cell cultures using a DNeasy Blood and Tissue Kit automated on a QIAcube (Qiagen) and then sequenced using the Illumina HumanOmni2.5M+Exome array at the Genetic Resources Core Facility (GRCF), Johns Hopkins, using standard Illumina protocols. This chip includes coverage of SNPs with a minor allele frequency (MAF) >2.5% in the 1000 Genomes Project pilot data. Illumina GenomeStudio ® software (version 2011.1, Genotyping module 1.9.4, Gentrain version 1.0) was used to analyze and call variants. The data was clustered using the Illumina standard.egt for the HumanOmni2-5Exome-8-v1-1- A array. All non-Y SNPs with a call rate of<85% were dropped. In addition, all Y and mitochondrial SNPs were manually reviewed, and edited or dropped as appropriate. All matched DNA samples were from either primary or immortalized cells (same patient). Control DNA was extracted from the whole blood of a non-affected, male human donor (Coriell Institute, Camden, New Jersey). For the Copy Number Variation (CNV) plots, segmentation analysis was performed on each chromosome / cell line to identify aggregate regions of copy-number gain or loss. We applied the Circular Binary Segmentation algorithm^18^ to the Log2Ratio data, as output from Genome Studio, and merged similar segments that were less than 2 sds, resulting in a median 1817 segments per sample.

### RNAseq

Prior to sequencing, total RNA was extracted from cultured cells using a RNeasy Kit (Qiagen) and the quality was checked on a Fragment Analyzer (Advanced Analytical Technologies, Inc.). The following workflow was followed to generate libraries for sequencing: 0.1-1 ug Total RNA was exposed to RiboZero Depletion and RNA Fragmentation. After the syntheses of first strand cDNA, the second strand cDNA was produced, followed by purification using AMPure XP beads. After 3’ end adenylation, adapter ligation and a 2^nd^ step of purification were executed. 15 cycles of PCR amplification were then employed, followed by a 3^rd^ step of purification. The library was validated using the Agilent Bioanalyzer High Sensitivity HS and Qubit. Paired-end 2x100 bp RNA-sequencing was carried out in the cell lines on the Illumina HiSeq2500. Reads were quantified with Kallisto^19^ aligned to Gencode^20^ v24 gene annotations.

### Whole Exome Sequencing

DNA from the cells was prepared for sequencing using the Agilent SureSelect Human All Exon V6 Kit as follows. 1 ug of genomic DNA was sheared using the Covaris E210 instrument (Covaris), shear time was decreased to 80 s in order to obtain larger insert sizes. A hybrid protocol for library preparation and whole exome enrichment was developed at CIDR (unpublished) based on methods and parameters from ref. 21, applied to the reagents, volumes and parameters from the Agilent SureSelect XT kit and automated protocol (p/n G7550-90000 revision B). All processing was done in 96 well plate formats using robotics (Beckman F/X, Perkin-Elmer Multiprobe II, Agilent Bravo, Beckman Biomek 2000). ‘With Bead’ clean ups were used following shearing, end repair, A-tailing and adapter ligation. The initial input of Ampure XP SPRI (Beckman Coulter Genomics) beads was based on volumes from the Agilent protocol. After the first clean up the sample was eluted and the beads remained in the reactions through the final ligation clean-up. These reactions were carried out using the XT reagent kits, volumes and conditions described in the Agilent protocol. At pre-capture PCR the entire product was amplified, adjusting the water in the reaction to accommodate the increase in DNA sample volume. The PCR enzyme used in all steps was switched from Herculase to Kapa Biosystems HiFi HotStart Ready Mix. The Kapa enzyme was used to increase coverage in GC rich regions. The number of PCR cycles was increased from 6 to 8 cycles. 750 ng of amplified library was used in an enrichment reaction following Agilent protocols (24 h hybridization). Post-capture washing was done using the Agilent protocol except the ‘off-bead’ catch process from ref. 21 was incorporated (samples are not eluted off the DynaBeads (Invitrogen), instead post-capture PCR master mix and indexes are added directly to the beads). Post-capture PCR was done according to the Agilent protocol, with the adjustment of water volume and PCR cycles where needed. For low input samples, 50 ng of genomic DNA was sheared using the Covaris E210 instrument (Covaris), using the same parameters as the 1 ug input samples. Library prep was performed using the Kapa Hyper Prep kit (Kapa Biosystems) according to the manufacturer’s protocol. Indexed adapters, primers and blockers used in the low input protocol were custom synthesized by Integrated DNA Technologies and were used in accordance to the Kapa protocol specifications. The hybridization for capture was set up according to the Agilent XT protocol with the exception that blocker #3 was exchanged for the IDT custom synthesized blocker. Post-capture washing was done using the Agilent protocol except the ‘off-bead’ catch process from^21^ was incorporated (samples are not eluted off the DynaBeads (Invitrogen), instead post-capture PCR master mix are added directly to the beads). Post-capture PCR was done according to the same parameters used for the pre-capture PCR adjusting for PCR cycles.

Libraries were sequenced on the HiSeq2500 platform with onboard clustering using 100 bp paired end runs and sequencing chemistry kits TruSeq Rapid v2 PE Cluster Kit-HS and TruSeq Rapid v2 SBS-HS.

Intensity analysis and base calling were performed through the Illumina Real Time Analysis (RTA) software (version 1.17.20). Base call files were demultiplexed from a binary format (BCL) to single sample fastq files using a demultiplexer written at CIDR as part of CIDRSeqSuite version 6.1 (unpublished).

Fastq files were aligned with BWA mem version 0.7.8 to the 1000 genomes phase 2 (GRCh37) human genome reference^22,23^. Duplicate molecules were flagged with Picard version 1.109(1716). Local realignment around indels and base call quality score recalibration were performed using the Genome Analysis Toolkit (GATK)^24^ version v3.1-1-g07a4bf8 or v3.3-0-g37228af. GATK’s reference confidence model workflow was used to perform joint sample genotyping (v3.3-0-g37228af). Briefly this workflow entails; 1) Producing a gVCF (genomic VCF) for each sample individually using HaplotypeCaller (--emitRefConfidence GVCF) for all bait intervals to generate likelihoods that the sites are homozygote reference or not 2) Joint genotyping the single sample gVCFs together with GenotypeGVCFs to produce a multi-sample VCF file. Variant filtering was done using hard filtering. Details on this filtering can be found in the included VariantFiltration script.

Single sample.vcf files were created from the multi-sample.vcf file. Variants that passed filtering were annotated using Annovar (version 2013_09_11) against a variety of data sources (dictionary file).

### Cell Growth Rate Measurements

For the 384-well plates, cell growth measurements were assessed by dispensing 35 μl of pNF cell suspensions into a black bottom Corning 3712 384-well assay plate (Corning, NY) at varying cell seeding densities (2000, 3000 and 4000) using a Multidrop Combi Reagent dispenser and a small pin cassette (Thermo Scientific, Fisher Scientific, Fair Lawn, NJ, USA). Assay plates were then placed into an IncuCyte Zoom (Essen Bioscience) where phase-contrast images were taken every two hours for a total of five days at 37 °C, 5% CO_2_, and 95% relative humidity. The Incucyte Zoom software calculated a % confluence as a Phase Object Confluence (percent). Growth rate values expressed as doubling times were determined by fitting the % Phase Object Confluence data points between time 0 h to whichever time the cells reached 80% confluency to the exponential growth function in GraphPad Prism 7 software.

Growth rates of the cells for the 1536-well plates were assessed using an endpoint cell viability assay (the Incucyte platform is not 1536-well compatible) as follows. 5 μl of pNF cell suspensions were dispensed into a white Corning 1536-well assay plate (Corning, NY) at cell seeding densities ranging from 250 to 1250 cells per well using a Multidrop Combi Reagent dispenser and a small pin cassette (Thermo Scientific, Fisher Scientific, Fair Lawn, NJ, USA). The plates were then covered with stainless steel Kalypsys lids and placed into an incubator at 37˚C, with 5% CO2 and 95% relative humidity. The plates were incubated for 24, 48 and 72 h and then 3 μl of CellTiter-Glo® assay from Promega (Madison, WI, USA) was added using a BioRAPTR® (Beckton Coulter, Brea, CA, USA). Plates were incubated for 30 min at room temperature, and relative luciferase units (RLU) were quantified using a ViewLux (PerkinElmer, Waltham, MA). Growth rate values expressed as doubling times were determined by fitting the data points to the exponential growth function in GraphPad Prism 7 software.

### Quantitative high-throughput (qHTS) drug screening and sensitivity analysis

All cells were screened with the MIPE-oncology compound library 4.0 (described above and syn4939906)^17^. The stocks are stored as 10 mM solutions in 100% DMSO at −80° C. syn49399062 lists individual compounds, mechanisms of action, stage of development, structure, and acquisition information. Information on the target(s) of the compounds was obtained by manual curation from publically available sources.

For the screening assay, 5 μl of pNF cell suspensions were dispensed into each well (except column 1, where the same volume of media without cells was added) of a white Corning 1536-well assay plate (Corning, NY) at a cell seeding density of 750 cells per well using a Multidrop Combi Reagent dispenser and a small pin cassette (Thermo Scientific, Fisher Scientific, Fair Lawn, NJ, USA). The compounds in the MIPE library were then transferred to columns 5 to 48 in a 1536-well assay plate (TC flat-bottom Greiner plates Catalog number: 789173-F) using a Kalypys pintool dispenser^25^. Controls were added to columns 1 through 4 of the assay plate. Column 1 contained media only, with DMSO, whereas columns 2 and 3 contained the proteosome inhibitor, Bortezomib, at a final concentration of 9 μM, and column 4 contained cells with DMSO. Each compound was tested at 11 concentrations, diluted 3-fold over a 4 log range, from 46 μM – 0.7 nM, at a final DMSO concentration of 0.4%. Dose responses of the compounds were intraplate along rows. The plates were then covered with stainless steel Kalypsys lids and placed into an incubator at 37˚C, with 5% CO2 and 95% relative humidity for 48 h, after which 3 μl of CellTiter-Glo® assay from Promega (Madison, WI, USA) was added using a BioRAPTR® (Beckton Coulter, Brea, CA, USA). Plates were incubated for 30 min at room temperature, and RLU were quantified using a ViewLux (PerkinElmer, Waltham, MA). RLU for each well were normalized to the median from the DMSO control wells as 100% viability (column 4), and median from control wells with media only as 0% viability (column 1).

Well level data was processed using in-house software that implements a grid-based curve fitting algorithm^26^. The resultant curves are characterized by four parameters – log potency (LAC50), the Hill slope and the two asymptotes. In addition, the response at the maximum concentration (MAXR), and a heuristic curve classification (CCLASS2) that generates CRC values for each compound were also calculated. The curve classification takes into account various features of the curve fit such as correlation to the observed responses, the presence or absence of asymptotes and so on. The resultant classification allows us to identify good quality curves (well defined asymptotes with full efficacy), inconclusive curves (partial asymptotes, partial efficacy) and inactive compounds (no concentration response). See Southall *et al.*^26^ for a detailed discussion on the curve classification scheme. For inactive compounds, a curve fit is usually not possible. However, in some cases a curve may be fit, but the CCLASS2 will flag it as an inactive curve. In such a case, the curve fit parameters should be ignored. CRC values of −1.1, −1.2, −2.1, and −2.2 are considered high quality hits; CRC values of −1.3, −1.4, −2.3, −2.4, and −3 are inconclusive hits, and a CRC value of 4 is for inactive compounds. An Area under the Curve (AUC) is also calculated using the trapezoidal method^27^ applied to the dose responses. The AUC is a useful measure since it implicitly incorporates potency and efficacy. Processed qHTS data parameter for each compound screened, in each cell line, can be found at: https://tripod.nih.gov/matrix-client/?p=562.

### Code availability

All data to perform the technical validation of the data described below can be found at https://github.com/Sage-Bionetworks/NTAP. Code for curve fitting can be obtained from https://tripod.nih.gov/curvefit/

## Data Records

### Data availability overview

All of the integrated data associated with this resource can be downloaded at http://www.synapse.org/pnfCellCulture (Data Citation 2). This resource serves as a landing page for the genomics data generated by the GRCF (Data Citation 3) as well as the drug sensitivity data collected by NCATS (https://tripod.nih.gov/matrix-client?p=562; Data Citation 1). RNA-Seq and Exome-Seq data is also available at the NCBI Sequence Read Archive (Data Citation 4).

To download data from Synapse requires acquiring a Synapse account at http://www.synapse.org/register using an email address. Data is freely available for download as well as exploration via interactive tools.

### Metadata collection and dictionary

Data has been annotated using a standard metadata dictionary that includes cell line name, assay and disease-related terms. These metadata can be used to query the data through the file views.

## Technical Validation

### SNP Array

**Figure 2:**
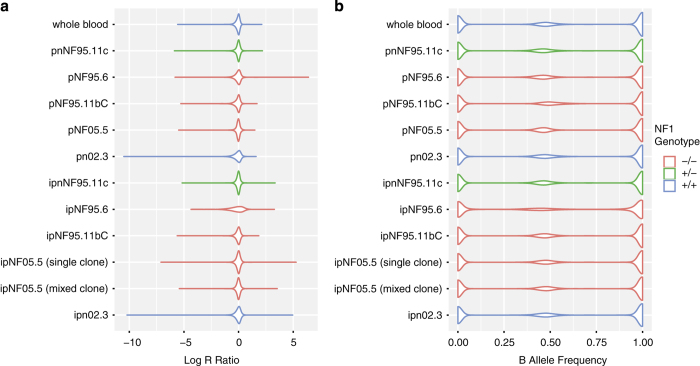
Distribution of (a) Log R Ratio values and (b) B Allele frequency values across the pNF cell lines.

To ensure that the SNP array values were sound we plotted the distribution of Log R Ratios and B Allele Frequencies of each sample measured. These values can be observed in Fig. 2.

### RNAseq

**Figure 3:**
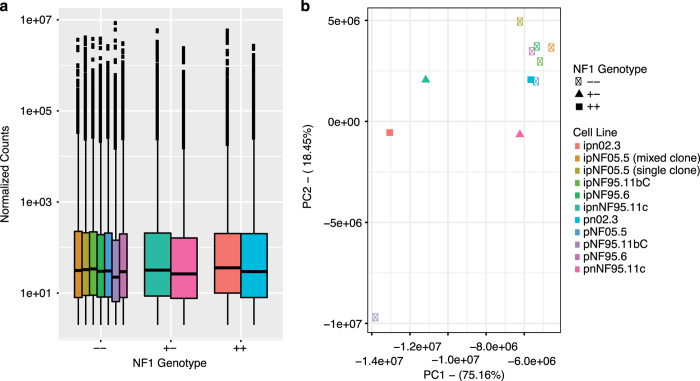
Distribution of RNA-Seq counts. (**a**) Boxplot representing normalized RNA-Seq counts across samples. **(b**) Principal component analysis (PCA) of samples.

We compared the counts of each RNAseq experiment to identify any outliers as depicted in Fig. 3a. The number of reads from sample pNF95.11bC were lower than the other samples, suggesting that this experiment was of lower quality. We also used principal component analysis to plot the samples in two dimensions as depicted in Fig. 3b.

### Whole Exome Sequencing

Sample quality control was carried out via 2% gels, OD260 readings and volume checks were done upon sample receipt at CIDR to confirm adequate quantity and quality of genomic DNA. In addition, samples were processed with an Illumina HumanCoreExome-24v1-0 array to confirm gender, identify unexpected duplicates and relatedness, confirm study duplicates and relatedness, provide sample performance information and sample identity confirmation against the sequencing data.

**Figure 4:**
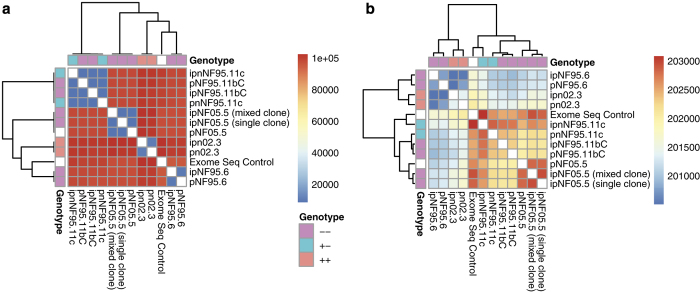
Clustering of variants in Whole Exome Sequencing data (a) represents discordance between samples and (b) represents the number of shared sites between samples.

We compared variants measured across all samples using bcftools^28^. Figure 4 depicts the outcome, with sample discordance shown in panel A and number of shared sites shown in panel b.

### Growth Rate Assessment

Although every cell line showed similar growth rate up to 80% confluency and slow or no growth if too dilute or over confluent, the recommended optimal splits between passages as well as the need for additional factors varied between cells are described here: ipn02.3 2λ and ipNF95.11b C split 1:4; ipNF05.5 and ipNF05.5 (Mixed clone); split 1:3, and Laminin matrix to help cells attach and grow better; ipNF95.6, split 1:3; and ipnNF95.11C, split 1:3 and if cell division slowed, neuregulin (glial growth factor 2, 20 ng/ml), a Schwann cell mitogen, was added.

**Figure 5:**
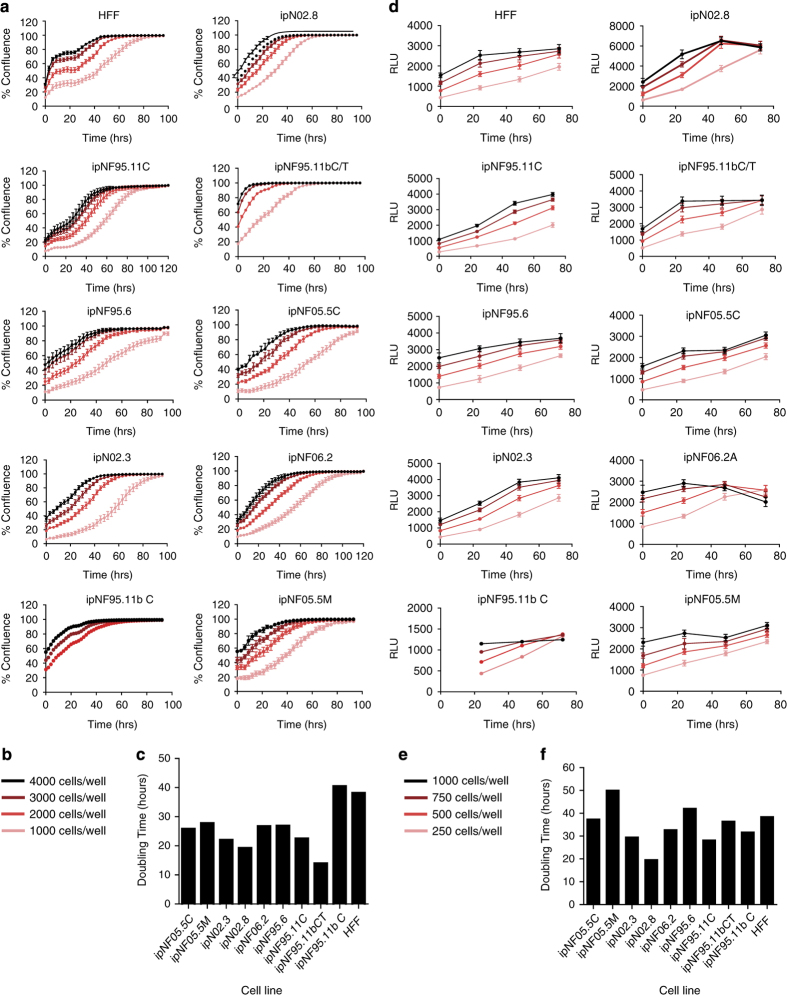
Growth rates for each cell line were determined in HTS microtiter well plate format. (**a**) Cell growth plots in 384-well plates as determined by phase contrast using an Incucyte Zoom, at different cell seeding densities. Percentage cell confluence was calculated by the software from the Incucyte Zoom instrument from the phase contrast signal. (**b**) Cell density legend for panel **a**. (**c**) Doubling times for each cell in a 384-well calculated by fitting an exponential growth curve on the data points from time 0 h to 80% confluence, using Graphpad Prism 7. Values are for curves obtained from seeding 2000 cells/well. (**d**) Cell growth plots in 1536-well plates as determined by CellTiterGlo luminescence signal, at different cell seeding densities, at different time points. (**e**) Cell density legend for panel **d**. (**f**) Doubling times for each cell in a 1536-well calculated by fitting the RLU at each time point to an exponential growth curve, using Graphpad Prism 7. Values are for curves obtained from seeding 250 cells/well.

Analysis and graphical representations of cell growth in 384-well assay plates were obtained using IncuCyte Zoom Software. Phase object confluence percentage verses hours is illustrated in Fig. 5a. Growth rate values expressed as doubling times were determined from the phase object confluence percentage data using GraphPad Prism 7 software, using the exponential growth function analysis. The cell growth rate plots for the 384-well plate are depicted in Fig. 5a and the calculated doubling times in Fig. 5c. Panels D and F of Fig. 5 show the growth rates and doubling times for the 1536-well plates respectively (measured by metabolic readout). In general, doubling times in 384-well were half of those in 1536-well format (median for all the cells tested were 27 and 53 h, for 384-well and 1536-well, respectively)^14^. The differences can be attributed to different measurement method (confluence by phase contrast versus metabolic readout) and effect of well size on growth rate of cells. The median doubling time calculated for these cells on a 12-well plate was 34 h, closer to those in 384-well plate^14^.

### Cell Proliferation Assay and Drug Screen

**Figure 6:**
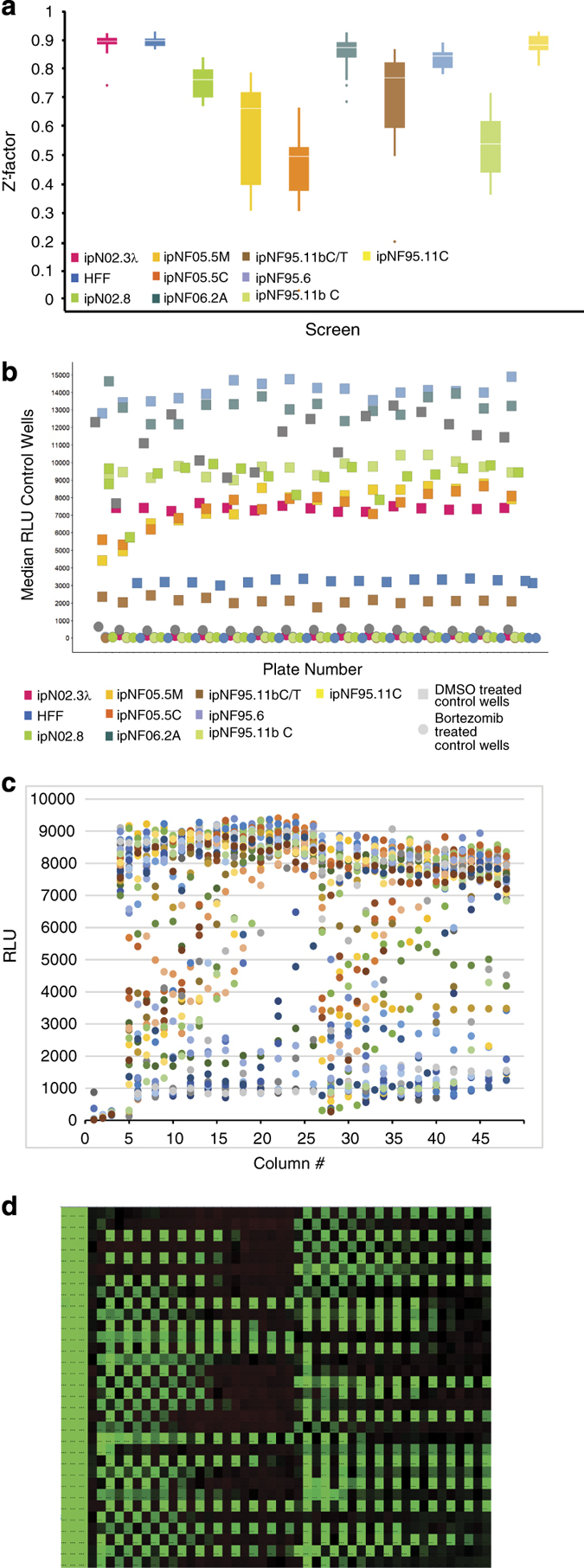
Quality control data for the dose response, quantitative high thoughput screen (qHTS) of the plexiform cells. (**a**) Box plots of Z′-factor for all plates in each screen. (**b**) Median RLU signal for column 4 (100% viability) and column 2 (pharmacological inhibition with 9 μM Bortezomib) for each plate of each screen. Colors reflect individual cell lines, as describe in the inset legend. DMSO treated control wells and 9 μM Bortezomib treated control wells (**c**) Scatter plot and (**d**) heat map of an assay plate from one of the screens. For the scatter plot, the X-axis corresponds to column number, and the Y-axis corresponds to relative luminescence units (RLU). Dots are coloured by plate row. For the heat map plot, the X-axis corresponds to column number, and the Y-axis corresponds to row number, and the colour coding is red, black, and green for increase, no change, and decrease, respectively, in RLU signal. Column 1 are wells with media only as low signal control (0% viability); columns 2 and 3 are wells with cells and 9 μM Bortezomib as pharmacological control, and column 4 are wells with cells and DMSO, as high signal control (100% viability).

The cell seeding density for the cell proliferation assay used for screening for all cells was chosen to be 750 cells/well based on the two criteria: linear growth rate for up to 48 h in 1536-well plate and robust number of RLU counts (>2000 RLU) at 48 h. The length of the compound incubation was chosen to be 48 h to allow for one or two cell doublings, and minimize evaporation from the wells and thus prevent edge effects on the data. The robustness of the 1536-well cell proliferation assay was assessed using the Z′-factor, which was calculated for each plate using the wells with the controls: wells in column 1 were used as low signal control and wells in column 4 were used as high signal control. The Z′-factor for each plate was calculated with the formula Z′-factor=1-3*(MAD column1+MAD column 4)/(Median column 4 - Median column 1), where MAD is the median absolute deviation, calculated using the formula MAD= median(|Xi-median(X)|)^29^. As described in Zhang *et al.*, 1999 (ref. 30), a Z′-factor>0.5 indicates that an assay is robust for HTS. The Z′-factors for each plate of each cell line screen are shown in Fig. 6a as a box plot, indicating that on average, the Z′-factors were >0.5 for each screen. Figure 6b depicts a scatter plot of the median of the column 3 (column with a Bortezamib, a pharmacological control) and 4 (column with cells and DMSO treatment, 100% activity control), showing how the signal (as RLU) was stable for each plate of each cell line screen. Figure 6c and d show plots from an actual assay plate from one of the screens. Figure 6c is a scatter plot of RLU (Y-axis) for each well of the assay plate (column number on X-axis). Figure 6d shows a heat map of the same assay plate, where row number is on the Y-axis and column number is in the X-axis. Colour coding is red, black, and green for increase, no change, and decrease, respectively, in RLU signal. Columns 1-4 correspond to control columns (see Material and Methods for plate map) and columns 5-48 include the compounds. The effect of some compounds in an intraplate dose response manner along wells in a row are clearly distinguished, and the overall lack of edge effects on the plates is evident. The primary qHTS of the MIPE collection on each cell line was done as N=1 given the robustness of the assay by Z′-factor and the implementation of dose response screening for each compound. The human foreskin fibroblast (HFF) was included as a non-tumor, non-Schwann control cell line for comparison of pharmacological activities.

## Usage Notes

### Data access and navigation

There are multiple ways to access the data described here. The primary site for the links to all data can be found at http://www.synapse.org/pnfCellCulture (Data Citation 2), which you can use to 1) download data via a web interface, 2) access the data via programmatic (R, Python, Java) tools or 3) query the data using an interactive app. Programmatic access can be achieved using the Synapse clients (https://github.com/Sage-Bionetworks/synapsePythonClient, https://github.com/Sage-Bionetworks/rSynapseClient/) using the synapse accession numbers and documentation for these clients is available at http://docs.synapse.org.

RNA-Seq and Exome-Seq data has also been uploaded to the Sequence Read Archive and is available for download at: (Data Citation 4).

Many of the cell lines that were characterized were profiled by the high-throughput drug screen (https://tripod.nih.gov/matrix-client?p=562) and with processed qHTS data parameter for each compound screened, in each cell line, can be found at: https://tripod.nih.gov/matrix-client?p=562 and at Synapse (Data Citation 1).

### Data Visualization

For specific queries on a single gene or drug of interest, we provide an online interface allowing interactive exploration of the data, available from the primary cell culture page (Data Citation 2) by clicking on the ‘pNF Cell Line Data Explorer’ link on the left side.

### Querying Synapse

All data are annotated using a consistent metadata dictionary and as such can be queried using one of the Synapse programmatic interfaces described above. For example, to query the Genomics data for RNASeq data from cell lines of a specific *NF1* genotype, we can use the following query at the command line:

synapse query "select * from syn8518944 where nf1Genotype='+/-' AND assay='rnaSeq'"

The same query in the Python client would look like this:

import synapseclient

syn = synapseclient.login()

results = syn.tableQuery(“select * from syn8518944 where nf1Genotype='+/-' AND assay='rnaSeq'")

And the R client:

library(synapser)

synLogin()

results <- synTableQuery(“select * from syn8518944 where nf1Genotype='+/-' and assay='rnaSeq'")

## Additional information

**How to cite this article:** Ferrer, M. *et al.* Pharmacological and genomic profiling of neurofibromatosis type 1 plexiform neurofibroma-derived schwann cells. *Sci. Data* 5:180106 doi: 10.1038/sdata.2018.106 (2018).

**Publisher’s note:** Springer Nature remains neutral with regard to jurisdictional claims in published maps and institutional affiliations.

## Supplementary Material



## Figures and Tables

**Table 1:** Samples, subjects, and data outputs (required).

**Sample Name**	**Sample Origin**	**Sample Genotype**	Media*	**Cell Line Authentication**	**SNP Array Data**	**RNASeq Data**	**Drug Sensitivity Data**	**ExomeSeq Data**
whole blood	whole blood	++			x			x
ipNF95.6	pNF95.6	--	Immortalized	x	x	x	x	x
ipNF95.11bC	pNF95.11b	--	Immortalized	x	x	x	x	x
ipNF05.5	pNF05.5	--	Immortalized	x	x	x	x	x
ipNF05.5 (mixed clones)	pNF05.5	--	Immortalized	x	x	x	x	x
ipn02.3	pn02.3	++	Immortalized	x	x	x	x	x
ipnNF95.11c	pnNF95.11c	+-	Immortalized	x	x	x	x	x
pNF05.5	pNF05.5	--	Primary	x	x	x		x
pNF95.11bC	pNF95.11b	--	Primary	x	x	x		x
pNF95.6	pNF95.6	--	Primary	x	x	x		x
pn02.3	pn02.3	++	Primary	x	x	x		x
pnNF95.1	pnNF95.11c	+-	Primary	x	x	x		x
ipn02.8		++	Immortalized				x	
ipNF06.2 A		--	Immortalized				x	
ipNF95.6	pNF95.6	--	Primary	x				
ipNF95.11bC	pNF95.11b	--	Primary	x				
ipNF05.5	pNF05.5	--	Primary	x				
ipNF05.5 (mixed clones)	pNF05.5	--	Primary	x				
ipn02.3	pn02.3	++	Primary	x				
ipnNF95.11c	pnNF95.11c	+-	Primary	x				
pn97.4	pn97.4	++	Primary		x	x		
ipn97.4	pn97.4	++	Immortalized		x	x		
pnNF95.12B	pnNF95.12B	+-	Primary		x	x		
HFF	HFF	++	Immortalized			x		
pNF04.4	pNF04.4	--	Primary		x	x		
ipNF04.4	pNF04.4	--	Immortalized		x	x		
sipnNF95.12B	pnNF95.12B	+-	Immortalized		x	x		

*Primary cell culture media: DMEM with 10% fetal bovine serum supplemented with glutamine and neuregulin on a laminin matrix. Immortalized cell culture media: DMEM, high glucose, GlutaMAX™ with 10% Fetal Bovine Serum (FBS) and 1X Penicillin-Streptomycin.
